# Pneumosepsis survival in the setting of obesity leads to persistent steatohepatitis and metabolic dysfunction

**DOI:** 10.1097/HC9.0000000000000210

**Published:** 2023-08-09

**Authors:** Avnee J. Kumar, Chitra Parthasarathy, Hallie C. Prescott, Scott J. Denstaedt, Michael W. Newstead, Dave Bridges, Angela Bustamante, Kanakadurga Singer, Benjamin H. Singer

**Affiliations:** 1 Department of Internal Medicine, Division of Pulmonary and Critical Care, University of Michigan School of Medicine, Ann Arbor, Michigan, USA; 2 VA Center for Clinical Management Research, Ann Arbor, Michigan, USA; 3 Department of Nutritional Sciences, University of Michigan School of Public Health, Ann Arbor, Michigan, USA; 4 Department of Pediatrics, Division of Endocrinology, University of Michigan School of Medicine, Ann Arbor, Michigan, USA

## Abstract

As critical care practice evolves, the sepsis survivor population continues to expand, often with lingering inflammation in many organs, including the liver. Given the concurrently increasing population of patients with NAFLD, in this study, we aimed to understand the long-term effect of sepsis on pre-existing NAFLD and hyperglycemia.

**Methods:**

Male mice were randomized to a high-fat diet or a control diet (CD). After 24 weeks on diet, mice were inoculated with *Klebsiella pneumoniae* (*Kpa*). Serial glucose tolerance tests, and insulin and pyruvate challenge tests were performed 1 week before infection and at 2 and 6 weeks after infection. Whole tissue RNA sequencing and histological evaluation of the liver were performed. To test whether persistent inflammation could be reproduced in other abnormal liver environments, mice were also challenged with *Kpa* after exposure to a methionine-choline–deficient high-fat diet. Finally, a retrospective cohort of 65,139 patients was analyzed to evaluate whether obesity was associated with liver injury after sepsis.

**Results:**

After *Kpa* inoculation, high-fat diet mice had normalized fasting blood glucose without a change in insulin sensitivity but with a notable decrease in pyruvate utilization. Liver examination revealed focal macrophage collections and a unique inflammatory gene signature on RNA analysis. In the clinical cohort, preobesity, and class 1 and class 2 obesity were associated with increased odds of elevated aminotransferase levels 1–2 years after sepsis.

**Conclusions:**

The combination of diet-induced obesity and pneumosepsis survival in a murine model resulted in unique changes in gluconeogenesis and liver inflammation, consistent with the progression of benign steatosis to steatohepatitis. In a cohort study, obese patients had an increased risk of elevated aminotransferase levels 1–2 years following sepsis.

## INTRODUCTION

Obesity is a disease characterized by the expansion of adipose tissue with both adipose-specific and systemic inflammatory consequences.^1^ Obesity not only predisposes to medical conditions, such as diabetes, heart failure, and coronary artery disease, but also contributes to an increased risk of admission to the intensive care unit. Clinical management of critically ill obese patients is complex due to multiple factors, including altered drug metabolism and complicated airway management.^2^ Despite these complexities, some studies note obesity is associated with improved survival in patients hospitalized for sepsis.^3–7^ Importantly, these observations are limited by their reliance on elevated body mass index (BMI), which does not necessarily correlate with adiposity.^7^ Though these studies implicate a protective effect of obesity on survival, the longer term interactions of prior sepsis and obesity on metabolism, inflammation, and obesity-associated diseases (eg, fatty liver disease or insulin resistance) have not been well explored.

Sepsis survival is associated with increased long-term risk of new cardiovascular, lung, kidney, and brain dysfunction.^8^ Persistently altered systemic and tissue-specific inflammatory responses are hypothesized to contribute to these complications.^8–12^ The pathogenesis of sepsis is in part related to metabolic-associated molecular patterns.^13^ Type II diabetes, a metabolic condition, when paired with sepsis, is thought to result in immune dysregulation.^14^ Not only this, obesity itself can also result in organ inflammation.^15,16^ Acutely, data show that obesity alters the effects of sepsis on the immune system, metabolism, and subsequent risk for organ injury. For example, after acute sepsis obese mice show improved mortality but worse kidney injury and altered glucose metabolism in surviving animals compared with lean mice.^17^ Obesity and polymicrobial sepsis result in worsened cardiac function.^18^ Acute sepsis through cecal ligation and puncture (CLP) in obese mice is also associated with increased neutrophilic inflammation in the liver.^19^ However, as prior murine work has focused on acute time points after sepsis, the interaction of obesity and sepsis survival on obesity-related organ dysfunction remains understudied. In addition, 24% of patients hospitalized with sepsis are obese; thus, examining the relationship of obesity with long-term organ injuries in sepsis survivors is also clinically relevant.^3^

NAFLD is a common complication of obesity. NAFLD is a growing health concern. Steatosis has a prevalence of 65% in patients with class I and class II obesity and 85% in those with a BMI >40 kg/m^2^.^20^ NASH, the progression of NAFLD, can predispose to hepatocellular carcinoma, cirrhosis, and result in a need for liver transplantation. It has been widely hypothesized that “second-hit” inflammatory stimulus or multiple parallel pathogenic insults are required for NAFLD to progress to NASH.^21^ Prior studies have postulated that inflammatory stimuli that progress NAFLD to steatohepatitis include oxidative stress and bacterial endotoxins.^22,23^ The long-term posthospital management of sepsis survivors is an increasingly important health concern, and the intersection of sepsis survival, obesity, and NAFLD has not been explored.

Motivated by this literature, we sought to evaluate the long-term outcomes of NAFLD in obese pneumosepsis survivors. To model murine sepsis, we used an established antibiotic-treated model of *Klebsiella pneumoniae* (*Kpa*) pneumosepsis survival, which we combined with diet-induced obesity.^8^ In this model, we hypothesize that the metabolic syndrome (fatty liver disease, obesity, and hyperglycemia) followed by septic insult results in persistent metabolic dysfunction and long-term liver inflammation. We found a decrease in blood glucose after pneumosepsis in obese mice. However, rather than representing an improvement of insulin sensitivity, this lowered blood glucose reflected persistent liver dysfunction, with notable changes in pyruvate metabolism and associated liver inflammation and macrophage burden. Although inflammation was seen in high-fat diet (HFD)–fed *Kpa-*infected mice, there was no evidence of collagen upregulation or fibrosis on picrosirius red evaluation. Therefore, we used a nutrient-deficient HFD with 0.1% methionine and no added choline (methionine-choline–deficient high-fat diet [MCD-HFD]). We found that MCD-HFD mice infected with *Kpa* had upregulation of liver *Col3a1* expression. Finally, using a large cohort of human sepsis survivors, we hypothesized that patients with obesity have a higher risk of developing long-term liver injury. We found that 1–2 years after admission for sepsis, there was an increased risk of elevated alanine aminotransferase (ALT) levels in patients with class I and class II obesity. These findings are important as they highlight the effects of sepsis on the progression of underlying medical conditions.

## METHODS

### Mouse studies

Wild-type male C57BL/6J mice were purchased from Jackson Labs. All procedures involving animals were undertaken in strict accordance with the recommendations of the Guide for the Care and Use of Laboratory Animals by the National Institutes of Health. The study protocol was approved by the University Committee on the Use and Care of Animals at the University of Michigan (PRO00008999 and PRO00010712).

### Diet-induced obesity model

HFD was 60% kCal/fat (D12492, Research Diets Inc.). The control diet (CD) was 10% kCal/fat (D12450J, Research Diets Inc.). Diet was initiated between wean and 1.5 months of age. HFD or CD was continued through the period of infection until animals were euthanized. HFD and CD were weighed 3 times a week to determine food intake, which was then divided by the number of animals in each cage and by animal weight. Diet was given ad lib.

### Methionine-choline–deficient and high-fat diet model

MCD-HFD was 60% kcal/fat with 0.1% methionine and no added choline (A06071302, Research Diets, Inc.). MCD-HFD was initiated 2 weeks before infection and switched back to the CD at the time of infection. Diet was given ad lib.

### Pneumosepsis survivor model

Mice, anesthetized with xylazine and ketamine, were inoculated intranasally with *Kpa* or saline control using a pipette tip. *Kpa*, strain ATCC 43816, serotype 2, at 1×10^4^ colony forming units was used for all experiments. Seventy-two hours after inoculation, all mice were given 5 days of i.p. ceftriaxone at a dose of 75 mg/kg.^8^

### Metabolic testing

Mice were fasted for 6 hours before initiation of glucose tolerance tests. Then, fasting blood glucose was measured using an EvenCare G2 glucometer and associated test strips. Following this, mice were injected i.p. with 0.7 g/kg d-glucose. Blood glucose was subsequently measured 15, 30, 45, 60, 90, and 120 minutes after injection. Mice were fasted for 4 hours before initiation of insulin tolerance tests. Then, fasting blood glucose was measured. Following this, mice were injected i.p. with 1 U/kg insulin. Blood glucose was then measured 15, 30, 45, 60, 75, 90, 105, and 120 minutes after injection. Mice were fasted for 6 hours before initiation of pyruvate challenge tests. Then, fasting blood glucose was measured. Following this, mice were injected i.p with 2.5 g/kg sodium pyruvate. Blood glucose was then measured 15, 30, 45, 60, 90, and 120 minutes after injection.

### RNA isolation from liver

Mice were euthanized using CO_2_ inhalation. The liver was removed, sectioned, and snap-frozen in liquid nitrogen. The liver was stored in −80 °C. The liver was homogenized, and RNA was isolated in Trizol (Thermo-Fisher) according to the manufacturer’s instructions. Genomic DNA digestion and RNA clean-up were completed using the RNAEasy Kit (Qiagen). Isolated RNA concentration and purity were evaluated using the NanoDrop (Thermo-Fisher).

### QuantSeq. 3′ mRNA sequencing

RNA samples were sent to the University of Michigan Advanced Genomics Core for QuantSeq. The University of Michigan Advanced Genomics Core constructed libraries using the Lexogen QuantSeq. 3′ mRNA FWD with the UMI add-on kit. The library was then subjected to 50 paired-end cycles on the Illumina High-Seq. 6000. Quality control and sequence alignment were completed using Cutadapt (v2.3), FastQC (v0.11.8), and FastQScreen (v0.13.0). Reads were mapped to the reference genome GRCm38 (Ensembl).

### Differential gene expression analysis

Data were filtered, removing genes with <10 total counts in all samples. Two different differential expression analyses were performed. Pairwise expression analyses using DESeq. 2 were performed to evaluate the main effects of infection and HFD.^24^ To identify genes in which the expression in HFD *Kpa* mice differed from all other groups, a differential gene expression analysis was performed using baySeq, using a negative binomial generalized linear model [thresholds: linear fold change >1.5 or <−1.5, Benjamini-Hochberg FDR (Padj)]. baySeq was used to compare multiple groups within a 2-factor design.^25^ The gene expression data from this study are available in Gene Expression Omnibus (Accession Number: GSE231504).

### Quantitative PCR gene expression analysis

cDNA was isolated from liver RNA. cDNA was generated using a high-capacity cDNA reverse transcription kit (Applied Biosystems). Quantitative PCR (qPCR) was performed on cDNA samples using the StepOnePlus thermocycle and primers *Chil3* (encoding CHIL3 protein, also known as YM1) and *Col3a1* from Integrated DNA technologies (Supplemental Table S1, http://links.lww.com/HC9/A413). TaqMan Fast Advanced Master Mix 2x (CAT# 4444557) was used for duplex PCR, with a probe for β-actin. Relative quantification of mRNA levels was plotted as fold change compared with saline CD. Measurements were performed in duplicate.

### Colony forming unit assays

Snap-frozen lung and liver were homogenized in sterile PBS. Aerobic culture of serial lysate dilutions was performed at 37 °C on tryptic soy agar plates.

### Histology and immunohistochemistry

Livers were obtained at the time of euthanasia. The largest lobe was sectioned and placed in a 4% paraformaldehyde solution. Livers were then paraffin wax embedded and sectioned sequentially. Sections were deparaffinized and washed in TBS. Then, using 1% H_2_O_2_, endogenous peroxidases were quenched. Ag retrieval was completed in a citrate buffer and a 90 °C water bath for 30 minutes and then cooled to room temperature. Slides were blocked with TBS blocking buffer (10% NGS + 3% BSA + 1% glycine + 0.4% TX- 100 in TBS) and then incubated in primary antibody Rabbit anti-YM1+YM2/*Chil3* 1:200 (Abcam EPR 15263) overnight. Slides were subsequently washed in TBS, incubated in avidin-biotin complex reagent, washed, and then incubated in FITC tyramide (1:50, Perkin Elmer). Slides were then washed with 1:10000 DAPI, and a coverslip was placed with prolonged antifade mounting media.

Picrosirius red staining was performed by the University of Michigan Dentistry Core using Weigert hematoxylin followed by Picrosirius Red (Sirius red F3B (C.I. 35782) and Saturated picric acid solution). Hematoxylin and eosin stain was performed by the Dentistry Core using Epredia Modified Mayer Hematoxylin and Stat lab Eosin with phloxine B.

### Imaging

Fluorescent images were captured using a Zeiss Axioplan 2 (209656) and the objectives (plan-NEOFLUAR 20x/0.50, 1004.072). Twenty-five images were obtained at ×20 by a blinded reviewer, which was then evaluated for the presence or absence of YM1 positive aggregates. Picrosirius red was visualized on a Nikon E800 microscope with 4×0/0.75 objective under polarized light with maximal cross-polarization and captured by a Nikon DS-U3 camera.

### Human observational study

To evaluate whether obesity modified liver injury after sepsis, we evaluated a national cohort of patients hospitalized with severe sepsis or septic shock in the Veterans Affairs healthcare system from 2015 to 2018. This retrospective chart review was deemed exempt by the Institutional Review Board. Sepsis hospitalizations were identified by evidence of infection and acute organ dysfunction in the electronic health record consistent with the Centers for Disease Control and Prevention’s sepsis surveillance definition, as described.^26,27^ Liver injury was defined as a change in ALT. Patients with baseline liver function measured within 180 days prior to sepsis hospitalization were included. Patients with total bilirubin >4.5 in the 180 before hospitalization were excluded. To minimize incorrectly charted data, patients with weight <75 pounds, weight >500 pounds, height >84 inches, or height >50 inches were excluded (Supplemental Table S2, http://links.lww.com/HC9/A414). Baseline BMI was calculated using the lowest weight in the 30 days before hospitalization and the tallest height in the 2 years before hospitalization. BMI was stratified using World Health Organization categories with underweight (<18.5), normal (18.5 to <25), preobesity (25 to <30), obesity class 1 (30 to <35), obesity class 2 (35 to <40), and obesity class 3 (≥40).

Acute liver injury was defined as the highest ALT measured from the day of emergency department (ED) presentation +30 days being >40 and greater than a 50% increase from baseline ALT. Subacute liver injury was defined as the lowest ALT measured from day + 31 to + 180 following ED presentation being >40 and a >50% increase from baseline ALT. One-year liver injury was defined as the lowest ALT measured from day + 181 to +365 following ED presentation being >40 and greater than a 50% increase from baseline ALT. Two-year liver injury was defined as the lowest ALT measured from day + 366 to + 730 following ED presentation being >40 and greater than a 50% increase from baseline ALT. Mortality was also evaluated.

This portion of the study was reviewed and deemed exempt by the Ann Arbor VA Institutional Review Board. Adjusted odds ratios were generated from logistic regression models with BMI category predicting the outcome. Models include the following covariates for adjustment: age, sex, select diagnoses (diabetes complicated, diabetes uncomplicated, alcohol use disorder, and liver disease), individual systemic inflammatory response syndrome (SIRS) criteria, and individual acute organ dysfunctions (excluding liver). SIRS was defined as 2 or more of the following: temperature between 36 °C and 38 °C, heart rate >90 beats per minute, a respiratory rate >20 breaths per minute, OR PaCO_2_ <32 mm Hg, white blood cells >12,000 cells/mm^3^, OR <4000 cells/mm^3^, and OR >10% immature (band) forms.^28^

### Data analysis

Data were analyzed using GraphPad Prism. For experiments involving 2 factors (eg, diet and infection), comparisons between groups were made using 2-way ANOVA for the effect of diet and pneumosepsis. Time course data were analyzed using repeated measures ANOVA. Post hoc comparisons between groups were adjusted using the Dunn-Šidák correction. Categorical data (eg, the presence or the absence of inflammatory aggregates) were analyzed by Fisher exact test. Unless otherwise specified, *p*-value <0.05 represented with *, < 0.01 with **, < 0.001 with ***, and <0.0001 with ****.

## RESULTS

### Fasting and peak blood glucose during glucose tolerance test is reduced in obese mice 2 weeks after *Kpa* pneumosepsis

**FIGURE 1:**
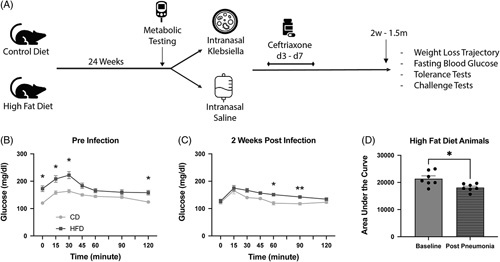
Pneumosepsis results in loss of fasting hyperglycemia and improved glucose tolerance in high-fat diet sepsis survivors. (A) Experimental design. Mice were started on a HFD or CD and underwent metabolic testing. At 24 weeks, mice were randomly allocated to intranasal *Kpa* or intranasal saline. After 72 hours, all mice received 5 days of ceftriaxone. Two weeks to 1.5 months later mice were sacrificed. (B) Presepsis, glucose tolerance test showed higher fasting hyperglycemia and higher peak glucose in HFD obese (n = 7) versus CD lean (n = 8) mice (repeated measures ANOVA, *p* < 0.001). (C) After sepsis, glucose tolerance test showed equivalent fasting and peak glucose in HFD obese (n = 7) versus control diet lean (n = 8) mice. However, there remained a significant difference among groups driven by differences at 60 and 90 minutes (repeated measures ANOVA, *p* < 0.01). (D) Two weeks after sepsis in obese mice, there was a significant difference in glucose tolerance preinfection (n = 7) and postinfection (n = 7). *p < 0.05 and **p < 0.01. Abbreviations: CD, control diet; HFD, high-fat diet; *Kpa*, *Klebsiella pneumoniae*.

Mice were started on CD or HFD, which was continued for at least 24 weeks, at which time metabolic testing was performed before infection. Mice were then anesthetized and then intranasally inoculated with *Kpa* or saline. All mice were then given 5 days of ceftriaxone, regardless of infection (Figure 1A). Antibiotic treatment resulted in effective clearance of infection, with no detectable bacterial dissemination to the liver at 14 days after inoculation (Supplemental Figure S1, http://links.lww.com/HC9/A487). Before infection, HFD mice had elevated fasting blood glucose compared with their CD counterparts, as expected. By repeated measures ANOVA, there was a significant difference between CD and HFD (*p* < 0.001) with principal differences at *t* = 0, 15, 30 minutes (*p* < 0.05, Figure 1B). Two weeks after pneumosepsis, after antibiotic therapy, HFD mice had similar blood glucose to the CD cohort (Figure 1C). In addition, when analyzing the AUC of glucose levels over time for individual mice, there was a significant decrease in blood glucose in response to glucose in HFD mice after infection compared with before infection (Figure 1D). Weight-based glucose dosing was completed using lean mass as determined by MRI findings. Of note, on MRI evaluation, there was no significant change in lean mass between groups 2 weeks after *Kpa* pneumosepsis (Supplemental Figure S2, http://links.lww.com/HC9/A417).

### Reduced fasting blood glucose in HFD-fed mice after *Kpa* pneumosepsis persists to 1.5 months, unrelated to change in weight or insulin tolerance

**FIGURE 2:**
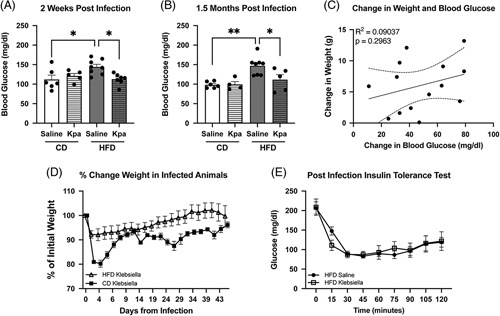
Improvement in fasting hyperglycemia in obese sepsis survivors persists to 6 weeks even with regain of baseline weight. There is no correlation between change in weight and change in blood glucose at 2 and 6 weeks and no change in insulin tolerance between infected and uninfected HFD obese mice. (A) Two weeks after infection, HFD obese-infected mice have significantly lower blood glucose compared with HFD obese–uninfected mice, on par with CD-uninfected lean mice. (B) 1.5 months after infection, HFD obese-infected mice have significantly lower blood glucose compared with HFD obese–uninfected mice, on par with CD-uninfected lean mice. (C) At 2 weeks and 1.5 months after infection, magnitude of change in weight (preinfection minus postinfection) is unrelated to the magnitude of change in blood glucose in HFD obese-infected mice. (D) HFD obese- and CD lean-infected mice initially lose weight, which they regain 1.5 months after infection. (E) insulin tolerance is the same in HFD obese-infected mice versus HFD obese-uninfected mice (repeated measures ANOVA, *p* = 0.97) *p < 0.05, and **p < 0.01. Abbreviations: CD, control diet; HFD, high-fat diet.

Given the notable change in blood glucose and AUC glucose tolerance testing, serial fasting blood glucose measurements were completed at 2 and 6 weeks after pneumosepsis. Two weeks after pneumosepsis, there was a significant decrease in fasting blood glucose in *Kpa* HFD mice compared with saline HFD mice (Figure 2A). This observation was not seen in mice-fed CD. Reduced fasting blood glucose persisted for 1.5 months after infection (Figure 2B). Both CD-fed and HFD-fed *Kpa* mice had decreased food intake up to 5 days after pneumosepsis. However, by the time of evaluation of fasting blood glucose, food intake had normalized between all groups for at least 7 days (Supplemental Figure S3, http://links.lww.com/HC9/A488). Taking data at both 2 weeks and 1.5 months after infection, specifically in *Kpa* HFD mice, the change in weight on the day of fasting blood glucose did not correlate with the magnitude of change in fasting blood glucose (Figure 2C). In addition, the *Kpa* HFD mice had regained 100% of baseline weight at 1.5 months after pneumosepsis (Figure 2D). To evaluate whether the change in glucose homeostasis was a simple result of increased insulin sensitivity specifically in HFD mice after infection, an insulin tolerance test was completed. There was no change in insulin tolerance in infected obese mice versus uninfected obese mice 2 weeks after infection (Figure 2E).

### Pyruvate utilization decreased in HFD mice compared with CD mice after *Kpa* pneumosepsis

**FIGURE 3:**
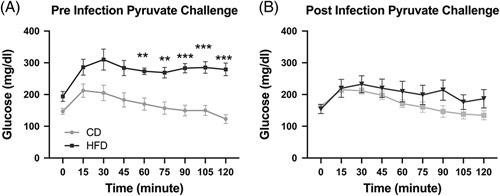
Surviving sepsis reduced pyruvate utilization in HFD obese mice compared with CD lean mice. (A) Presepsis, there is altered pyruvate metabolism in HFD obese mice compared with CD lean mice (repeated measures ANOVA, *p* < 0.001). (B) After sepsis, there is no statistically significant difference in pyruvate metabolism in HFD obese mice compared with CD lean mice (repeated measures ANOVA, *p* = 0.28). *p < 0.05, **p < 0.01, and ***p < 0.001. Abbreviations: CD, control diet; HFD, high-fat diet.

Given that there did not seem to be a change in insulin tolerance in HFD mice, regardless of infection, we turned our attention to liver gluconeogenesis. To explore whether liver gluconeogenesis is impaired after infection, a pyruvate challenge was performed in HFD obese versus CD lean mice infected with *Kpa*. Before infection, the pyruvate challenge resulted in higher peak blood glucose levels, as expected, in HFD animals (Figure 3A). After pneumosepsis, there was a change in pyruvate utilization in HFD mice (Figure 3B), with decreased glucose production in response to the pyruvate challenge.

### Dysregulated interferon signaling and innate immune responses in hepatic transcriptomic analysis of HFD mice after *Kpa* pneumosepsis

**FIGURE 4:**
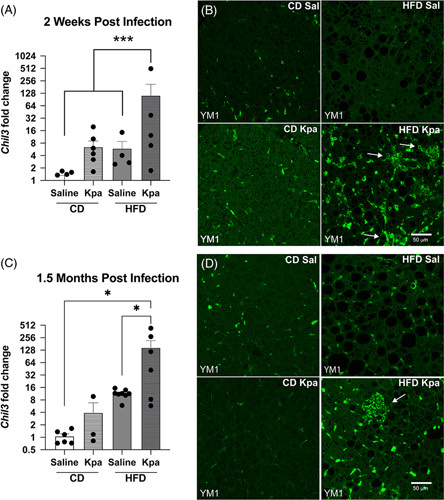
After sepsis, there is increased *Chil3*/YM1 in HFD obese mice by RNA and immunohistochemistry. (A) Two weeks after sepsis, there is significantly higher *Chil3* expression in HFD *Kpa* mice than in other groups (baySeq padj = 0.0006). (B) Two weeks after sepsis, YM1 positive aggregates are more common in the liver of HFD *Kpa* mice (HFD Kpa 2 mice without aggregates and 4 with aggregates; CD Kpa 7 mice without aggregates and 1 with aggregates; HFD Saline 5 mice without aggregates and 0 with aggregates; and HFD Saline 5 mice without aggregates and 0 with aggregates, Fisher Exact test *p* = 0.007). (C) 1.5 months after sepsis, there is significantly higher *Chil3* expression in HFD *Kpa* mice. (D) 1.5 months after sepsis, YM1 positive aggregates are seen in HFD *Kpa* mice. *p < 0.05, **p < 0.01, ***p < 0.001. Abbreviations: CD, control diet; HFD, high-fat diet; *Kpa*, *Klebsiella pneumoniae*.

Change in pyruvate response after pneumosepsis in HFD mice pointed to a change in liver function as an etiology of decreased blood glucose, and thus, RNA isolated from the livers was evaluated using 3′ end RNA sequencing (QuantSeq, with pairwise and Bayesian differential expression analysis available in Table S3, http://links.lww.com/HC9/A415). 216 genes were found to be differentially expressed specifically in the obese-infected mice compared to all other groups using baySeq (Supplemental Table S3, http://links.lww.com/HC9/A415). Gene ontology analysis revealed a number of differences, including pathways consistent with dysregulated interferon signaling and innate immune responses (Supplemental Table S4, http://links.lww.com/HC9/A416).^29^ In addition, we found several markers of macrophage activation. Specifically, in our baySeq analysis, *Chil3* (encoding CHIL3 protein, also known as YM1) was increased in HFD *Kpa* survivors at 2 weeks following infection (Figure 4A). YM1 is notably known to mark macrophages associated with the development of NASH.^30^ We sought to validate our RNA sequencing using immunohistochemistry (IHC). Hematoxylin and eosin staining revealed steatosis as expected in HFD animals (Supplemental Figure S5, http://links.lww.com/HC9/A490). Immunohistochemistry staining for YM1 showed aggregates specifically in the livers of *Kpa* HFD mice (Figure 4B) (HFD Kpa 2 mice without aggregates, 4 with aggregates; CD Kpa 7 mice without aggregates, 1 with aggregates; HFD Saline 5 mice without aggregates, 0 with aggregates; and HFD Saline 5 mice without aggregates, 0 with aggregates, Fisher exact test *p* = 0.007). Increased *Chil3* expression persisted for 1.5 months after *Kpa* survival (Figure 4C). At 1.5 months, immunohistochemistry for YM1 showed the persistence of aggregates in the livers of *Kpa* HFD mice (Figure 4D).

### *Kpa* pneumosepsis is associated with enhanced liver inflammation and fibrosis in a model of diet-induced NASH

**FIGURE 5:**
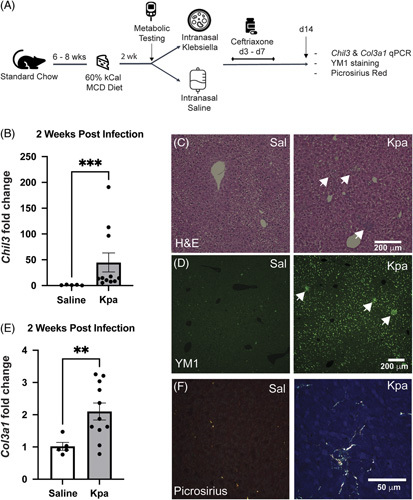
Klebsiella infection in MCD-HFD mice results in increased *Chil3* and *Col3a1* expressions, with the concurrent increase in picrosirius red staining. (A) Experimental design. At 6–8 weeks of age, mice were started on an MCD-HFD for 2 weeks and underwent metabolic testing. Then, mice were randomly allocated to intranasal *Kpa* or intranasal saline. After 72 hours, all mice received 5 days of ceftriaxone. Two weeks later, mice were sacrificed. (B) Two weeks after sepsis, there is significantly higher *Chil3* expression in MCD-HFD *Kpa* mice. (C) Two weeks after sepsis, MCD-HFD *Kpa* mice have aggregates seen in the liver parenchyma on H&E. (D) Two weeks after sepsis, MCD-HFD *Kpa* mice have YM1 aggregates scattered throughout the liver parenchyma on IHC. Aggregates and heavy sinusoidal staining were present in significantly more MCD-HFD *Kpa* livers (8 with, 3 without) than with MCD-HFD alone (0 with, 5 without, and Fisher Exact test *p* = 0.02). (E) Two weeks after sepsis, there is higher liver *Col3a1* expression in MCD-HFD *Kpa* mice. (F) Two weeks after sepsis, parenchymal picrosirius red staining by polarized light is evident in *Kpa* MCD-HFD mice. **p < 0.01, ***p < 0.001. Abbreviations: H&E, hematoxylin and eosin; HFD, high-fat diet; IHC, immunohistochemistry; *Kpa*, *Klebsiella pneumonia*; MCD, methionine-choline–deficient.

Given the evidence of ongoing steatohepatitis and associated macrophage activation in the livers of *Kpa* HFD mice, we sought to evaluate whether this was associated with differences in hepatic fibrosis using liver *Col13a1* expression and picrosirius red staining for evidence of type III collagen deposition. In our *Kpa*-treated HFD mice, there was no significant difference in *Col3a1* expression (Supplemental Figure S4, http://links.lww.com/HC9/A489). Furthermore, picrosirius red staining as evaluated by polarized light was negative in all 4 groups, indicating no development of fibrosis (data not shown). In murine models, HFD alone without nutrient deficiencies requires prolonged exposure to result in liver fibrosis. Therefore, to determine whether hepatic fibrosis could be observed more generally after pneumosepsis survival in a disordered liver microenvironment, we used another model that more closely mimics NASH by subjecting young (6–8 wk) mice to a 2-week pulse of MCD-HFD.^31^ Mice were first challenged with MCD-HFD before infection (Figure 5A). We found that, after infection, *Kpa*-infected mice showed an increase in *Chil3* gene expression (Figure 5B). On hematoxylin and eosin stained sections, there was an increase in aggregates in the parenchyma, which were found to be YM1 positive (Figure 5C, D). Notably, there was upregulation of *Col3a1* expression (Figure 5E), and picrosirius red by polarized light staining showed increased staining in *Kpa*-infected mice indicating the development of fibrosis (Figure 5F). These findings indicate generalizability to our results in HFD mice that prior pneumosepsis can augment liver-associated inflammation and contribute to fibrosis.

### Sepsis increases risk for liver injury 1–2 years after septic insult in humans

In a cohort study of patients hospitalized with severe sepsis or septic shock, there were 81,196 index hospitalizations, 65,139 of which had a measured baseline ALT, BMI, and total bilirubin <4.5 (Supplemental Table S2, http://links.lww.com/HC9/A414). Two years after index hospitalization, patients with preobesity, class 1, class 2, and class 3 obesity had greater survival (68%, 76%, 80%, and 82%, respectively) compared with patients with normal weight (56%). However, the OR for elevation in ALT increased significantly 1–2 years after index hospitalizations significantly in pre, class 1, and class 2 obesity (Table 1) but not class 3 obesity.

**TABLE 1:** Risk of chronic liver injury is elevated 1–2 years after sepsis in obese patients

BMI categories	OR for day 366–730 liver injury
Underweight	1.15 (0.78, 1.70)
Normal	Reference
Preobesity	1.30 (1.07, 1.58)^a^
Class 1 obesity	1.54 (1.25, 1.89)^a^
Class 2 obesity	1.30 (1.00, 1.68)^a^

*Note:* Including 65,139 hospital admissions for sepsis or septic shock from 2015 to 2018 in the Veterans Administration. ORs generated from logistic regression models with BMI category predicting the outcome. Models include the following covariates for adjustment: age, sex, select diagnoses, individual SIRS criteria, and individual acute organ dysfunctions (except liver). BMI, body mass index; SIRS, systemic inflammatory response syndrome.

a *p* < 0.05.

## DISCUSSION

Obesity, NAFLD, and sepsis survival are conditions with increasing worldwide prevalence. There is epidemiological data regarding the intersection of these conditions and, however, complicated by confounders in the care of patients with obesity. Sepsis and obesity have been specifically studied in murine models, usually in models of lethal sepsis.^32^ However, there is a paucity of murine data regarding sepsis survivorship, a clinical entity with known long-term multiorgan dysfunction.^33^

In this study, we aimed to understand the long-term effect of pneumosepsis survivorship on underlying obesity when associated with fatty liver disease. Surprisingly, pneumosepsis resulted in decreased fasting blood glucose in mice with underlying diet-induced fatty liver disease and hyperglycemia. Lowered fasting blood glucose can certainly be due to weight loss and a concurrent improvement in insulin sensitivity, as is seen in bariatric surgery.^34^ However, we found no difference in insulin sensitivity in obese-uninfected mice and obese pneumosepsis survivor mice.

Given the lack of difference in insulin sensitivity, we aimed to evaluate gluconeogenesis, as hepatic glucose production is a significant driver of glucose homeostasis in rodents.^35^ Specifically, pyruvate is key *in vivo* substrate of gluconeogenesis.^36^ Pyruvate is important to glucose metabolism and can be used in liver anabolic and catabolic processes.^37^ Therefore, we proceeded with a pyruvate tolerance test and notably found a significant change in pyruvate utilization in obese pneumosepsis survivor mice. This unique finding drew our attention to the histological and transcriptomic evaluation of the liver as potentially hepatic dysfunction explained improvement in blood glucose in obese *Kpa*-infected mice. Notably, the liver revealed increased inflammation, persistent to 1.5 months after infection.

Macrophages are a cardinal force in the development of fibrosis and inflammation. In obese states, macrophages can exhibit plasticity and cause the release of proinflammatory cytokines.^38^ In a prior murine model, fatty liver disease followed by sepsis through CLP time to mortality was attenuated by the deletion of TREM2 macrophages.^39^ In fibrotic murine livers, there is upregulation of both M1 and M2 macrophages. However, data show that, in livers undergoing resolution of fibrosis, there was a notable dearth of M2 macrophages, implying that the M1 macrophages were largely responsible for fibrosis resolution.^40^ The alternative macrophage pathway is also postulated to be a prevailing force in fibrogenesis.^41–43^ YM1, an M2 macrophage marker, has been used as a marker for differential upregulation in a murine NASH model.^30^ YM1 has also been used in evaluating lung fibrosis and is thought to potentially be a marker of oxidative stress.^44^

Given this signature of liver inflammation on pre-existing fatty liver disease after *Kpa* pneumosepsis, we postulated that pneumosepsis is the “second hit” that results in the development of steatohepatitis and potentially fibrosis. Sepsis stimulates free fatty acid production resulting in worsened metabolic disorders, and the free fatty acids can then cause inflammation and lipotoxic effects on the liver.^13^ Potentially oxidative stress and the release of free fatty acids in sepsis could impact the progression of NAFLD to NASH. In fact, a prior study showed the use of C75, a fatty acid synthase inhibitor, lowered free fatty acid accumulation in the liver, and increased survival after CLP.^45^ However, it is well known that murine models of steatohepatitis and fibrosis are difficult to elucidate on HFD alone without prolonged exposure, toxins, or provocative nutrient deficiencies, such as a methionine-choline–deficient diet. We aimed to intensify our infectious model with a highly inflammatory MCD-HFD to accelerate fibrosis. Therefore, we infected a group of mice after a 2-week exposure. In this cohort, we found upregulation of *Col3a1* and increased picrosirius red staining by polarized light indicating collagen deposition and fibrosis, in addition to the accumulation of YM1 expressing macrophages and increased *Chil3* expression.

This study focuses on sepsis survivorship, rather than acute infection. In this survival model of pneumosepsis, all animals regardless of infection receive antibiotics with the intention of clearing disseminated infection, rather than producing a state of low-level “chronic” sepsis.^8^ In this model, when mice are euthanized before antibiotic therapy, bacteria are cultured from the liver; however, after antibiotic therapy, bacteria are absent. This indicates that persistent inflammation is self-sustaining, rather than a response to chronic infection. Interestingly, although there is a concern in humans for “obese sarcopenia” or loss of lean mass during sepsis, in our model, we found that, by 2 weeks, obese sepsis mice had regained lean mass but not fat mass. The preservation of lean mass in our study contradicts prior sepsis work in a CLP model.^46^ However, the route of infection—intra-abdominal surgery to cause peritoneal sepsis versus intranasal inoculation to cause pneumosepsis—may account for this difference.

Early detection and treatment of sepsis have improved outcomes, therefore leading to an increased sepsis survivor population.^47^ Long-term outcomes are complicated by postintensive care syndrome and difficulty in returning to work exacerbated by socioeconomic inequalities.^48^ There are limited data on long-term liver outcomes after septic insult. Given this signature of liver inflammation after pneumosepsis in mice with pre-existing fatty liver disease, we looked to see if we saw a similar phenotype clinically. Abnormal transaminase levels (eg, ALT) are often the first clinically recognized marker of liver dysfunction in the outpatient setting and frequently trigger an evaluation for NASH in obese patients. As such, we sought to evaluate whether obese patients who survive sepsis are at increased risk of developing new abnormalities in liver transaminases. In our retrospective patient cohort, we showed, as in prior studies, that obesity is associated with improved survival in sepsis—consistent with the “obesity paradox.”^3^ Despite this survival advantage, obesity was associated with an increased risk of elevated ALT 1–2 years after hospitalization, raising the question of whether survival is marked by the progression of chronic liver disease in this population. Our human sepsis data were retrospective and lacked a confirmation of underlying fatty liver disease or other concomitant liver diseases. Nevertheless, this signal is intriguing, and we aimed to minimize these cofounders using within-person controls.

Our animal study certainly does have limitations. Although we performed multiple metabolic tests, the challenge and tolerance tests do not simultaneously measure variables in murine models as would be seen in hyperinsulinemic-euglycemic clamps. Our data are largely observational and do not use macrophage-specific knockouts to determine whether there is a reversal of persistent inflammation. Although our study is a survival model, and therefore required the use of antibiotics, the potential for changes to the microbiome and side effects of antibiotics exists. We mitigated this weakness by giving the same antibiotics to both infected and sham animals. Finally, we used a provocative MCD-HFD diet to determine whether there was evidence of a fibrotic signature. However, this diet could have differential metabolic effects given deficiencies in methionine and choline that are not fully understood.

Future studies on sepsis survivorship, NAFLD, and obesity should address potential therapeutic targets. As our population of patients with obesity increases, it is appropriate that our care and understanding increase. We should be able to offer precision medicine to patients across obesity classes. Not only does our system-wide support need to expand but we also need to understand how obesity modulates admissions for critical illness to provide patients with obesity exceptional clinical care.

## CONCLUSIONS

In a reproducible diet-induced obesity murine model, there is evidence of changes in gluconeogenesis and liver inflammation after septic insult in obese *Klebsiella* inoculated mice. Histologic changes after infection were further exacerbated with the use of an MCD-HFD. Furthermore, in a cohort study of patients admitted with sepsis, overweight and obese patients had an increased risk of elevated ALT 1–2 years following admission for sepsis. These retrospective data are limited as there is no confirmation of coexisting NAFLD or histological development of inflammation.

## FUNDING INFORMATION

The authors thank the support of the Charles Woodson Collaborative Research Award from the University of Michigan Department of Pediatrics. Benjamin H. Singer is supported by R01AG074968, and Kanakadurga Singer is supported by RO1DK115583. Scott J. Denstaedt is supported by K08HL153799. Hallie C. Prescott is supported by AHRQ R01 HS026725.

## CONFLICTS OF INTEREST

The authors have no conflicts to report.

## Supplementary Material

## References

[1] McGuire TR Brusnahan SK Bilek LD Jackson JD Kessinger MA Berger AM. Inflammation associated with obesity: Relationship with blood and bone marrow endothelial cells. Obesity. 2011;19:2130–6. 21901025 10.1038/oby.2011.246

[2] Schetz M De Jong A Deane AM Druml W Hemelaar P Pelosi P. Obesity in the critically ill: a narrative review. Intensive Care Med. 2019;45:757–69. 30888440 10.1007/s00134-019-05594-1

[3] Prescott HC Chang VW O’Brien JM Langa KM Iwashyna T. Obesity and one-year outcomes in older Americans with severe sepsis. Crit Care Med. 2014;42:1766–74. 24717466 10.1097/CCM.0000000000000336 PMC4205159

[4] Karampela I Chrysanthopoulou E Christodoulatos GS Dalamaga M. Is there an obesity paradox in critical illness? Epidemiologic and metabolic considerations. *Curr Obes Rep*. 2020;20:1–14. 10.1007/s13679-020-00394-x 32564203

[5] Wacharasint P Boyd JH Russell JA Walley KR. One size does not fit all in severe infection: obesity alters outcome, susceptibility, treatment, and inflammatory response. Crit Care Lond Engl. 2013;17:R122. 10.1186/cc12794 PMC4057102 23786836

[6] Ng PY Eikermann M. The obesity conundrum in sepsis. BMC Anesthesiol. 2017;17:147. 29070011 10.1186/s12871-017-0434-z PMC5657099

[7] Pepper DJ Demirkale CY Sun J Rhee C Fram D Eichacker P. Does obesity protect against death in sepsis? A retrospective cohort study of 55,038 adult patients. Crit Care Med. 2019;47:643–50. 30789403 10.1097/CCM.0000000000003692 PMC6465121

[8] Denstaedt SJ Spencer-Segal JL Newstead M Laborc K Zeng X Standiford TJ. Persistent neuroinflammation and brain specific immune priming in a novel survival model of murine pneumosepsis. Shock. 2019;54:78–86. 10.1097/SHK.0000000000001435 PMC7015772 31415473

[9] Denstaedt SJ Bustamante AC Newstead MW Moore BB Standiford TJ Zemans RL. Long-term survivors of murine sepsis are predisposed to enhanced LPS-induced lung injury and proinflammatory immune reprogramming. Am J Physiol Lung Cell Mol Physiol. 2021;321:L451–65. 34161747 10.1152/ajplung.00123.2021 PMC8410111

[10] Denstaedt SJ Spencer-Segal JL Newstead MW Laborc K Zhao AP Hjelmaas A. S100A8/A9 drives neuroinflammatory priming and protects against anxiety-like behavior after sepsis. J Immunol. 2018;200:3188–200. 29563178 10.4049/jimmunol.1700834 PMC5915914

[11] Yende S Kellum JA Talisa VB Peck Palmer OM Chang CCH Filbin MR. Long-term host immune response trajectories among hospitalized patients with sepsis. JAMA Netw Open. 2019;2:e198686. 31390038 10.1001/jamanetworkopen.2019.8686 PMC6686981

[12] Singer BH Newstead MW Zeng X Cooke CL Thompson RC Singer K. Cecal ligation and puncture results in long-term central nervous system myeloid inflammation. PloS One. 2016;11:e0149136. 26862765 10.1371/journal.pone.0149136 PMC4749127

[13] Zhu X Zhang W Wu C Wang S Smith FG Jin S. The novel role of metabolism-associated molecular patterns in sepsis. Front Cell Infect Microbiol. 2022;12:1–7. 10.3389/fcimb.2022.915099 PMC9202891 35719361

[14] Frydrych LM Fattahi F He K Ward PA Delano MJ. Diabetes and sepsis: risk, recurrence, and ruination. Front Endocrinol. 2017;8:271. 10.3389/fendo.2017.00271 PMC5670360 29163354

[15] Mancuso P. Obesity and lung inflammation. J Appl Physiol. 2010;108:722–8. 19875709 10.1152/japplphysiol.00781.2009 PMC2838639

[16] Rivera CA. Pathophysiology of obesity. Pathophysiol Off J Int Soc Pathophysiol ISP. 2008;15:69–70. 10.1016/j.pathophys.2008.04.006 PMC2605687 18583111

[17] Lewis ED Williams HC Bruno MEC Stromberg AJ Saito H Johnson LA. Exploring the obesity paradox in a murine model of sepsis: improved survival despite increased organ injury in obese mice. Shock. 2021;57:151–9. 10.1097/SHK.0000000000001856 PMC8678195 34482320

[18] DeMartini T Nowell M James J Williamson L Lahni P Shen H. High fat diet-induced obesity increases myocardial injury and alters cardiac STAT3 signaling in mice after polymicrobial sepsis. Biochim Biophys Acta Mol Basis Dis. 2017;1863(10 Pt B):2654–60. 28625915 10.1016/j.bbadis.2017.06.008 PMC5653424

[19] Kaplan JM Nowell M Lahni P Shen H Shanmukhappa SK Zingarelli B. Obesity enhances sepsis-induced liver inflammation and injury in mice. Obesity. 2016;24:1480–1488. 27172993 10.1002/oby.21504 PMC4925204

[20] Fabbrini E Sullivan S Klein S. Obesity and nonalcoholic fatty liver disease: biochemical, metabolic and clinical implications. Hepatol Baltim Md. 2010;51:679–89. 10.1002/hep.23280 PMC3575093 20041406

[21] Peng C Stewart AG Woodman OL Ritchie RH Qin CX. Non-alcoholic steatohepatitis: a review of its mechanism, models and medical treatments. Front Pharmacol. 2020;11:1864. 10.3389/fphar.2020.603926 PMC7745178 33343375

[22] Kessoku T Kobayashi T Imajo K Tanaka K Yamamoto A Takahashi K. Endotoxins and non-alcoholic fatty liver disease. Front Endocrinol. 2021;12:770986. 10.3389/fendo.2021.770986 PMC8586459 34777261

[23] Rivera CA. Risk factors and mechanisms of non-alcoholic steatohepatitis. Pathophysiol Off J Int Soc Pathophysiol ISP. 2008;15:109–14. 10.1016/j.pathophys.2008.04.003 PMC2600439 18667295

[24] Love MI Huber W Anders S. Moderated estimation of fold change and dispersion for RNA-seq data with DESeq. 2. Genome Biol. 2014;15:550. 25516281 10.1186/s13059-014-0550-8 PMC4302049

[25] Hardcastle TJ Kelly KA. baySeq: empirical Bayesian methods for identifying differential expression in sequence count data. BMC Bioinformatics. 2010;11:422. 20698981 10.1186/1471-2105-11-422 PMC2928208

[26] Wayne MT Molling D Wang XQ Hogan CK Seelye S Liu VX. Measurement of sepsis in a national cohort using three different methods to define baseline organ function. Ann Am Thorac Soc. 2021;18:648–55. 33476245 10.1513/AnnalsATS.202009-1130OC PMC8008999

[27] Hospital Toolkit for Adult Sepsis Surveillance. Centers for Disease Control and Prevention. 2018. Accessed November 2022. https://www.cdc.gov/sepsis/pdfs/sepsis-surveillance-toolkit-mar-2018_508.pdf

[28] Levy MM Fink MP Marshall JC Abraham E Angus D Cook D. 2001 SCCM/ESICM/ACCP/ATS/SIS International Sepsis Definitions Conference. *Crit Care Med*. 2003;31:1250–6. 12682500 10.1097/01.CCM.0000050454.01978.3B

[29] Ge SX Jung D Yao R. ShinyGO: a graphical gene-set enrichment tool for animals and plants. Bioinformatics. 2020;36:2628–9. 31882993 10.1093/bioinformatics/btz931 PMC7178415

[30] George J Zhang Y Sloan J Sims JM Imig JD Zhao X. Tim-1 deficiency aggravates high-fat diet-induced steatohepatitis in mice. Front Immunol. 2021;12:747794. 34675931 10.3389/fimmu.2021.747794 PMC8523998

[31] Radhakrishnan S Ke JY Pellizzon MA. Targeted nutrient nodifications in purified diets differentially affect nonalcoholic fatty liver disease and metabolic disease development in rodent models. Curr Dev Nutr. 2020;4:nzaa078. 32494762 10.1093/cdn/nzaa078 PMC7250583

[32] Frydrych LM Bian G O’Lone DE Ward PA Delano MJ. Obesity and type 2 diabetes mellitus drive immune dysfunction, infection development, and sepsis mortality. J Leukoc Biol. 2018;104:525–534. 30066958 10.1002/JLB.5VMR0118-021RR

[33] Gritte RB Souza-Siqueira T Curi R Machado MCC Soriano FG. Why septic patients remain sick after hospital discharge? Front Immunol. 2021;11:605666. 33658992 10.3389/fimmu.2020.605666 PMC7917203

[34] Pareek M Schauer PR Kaplan LM Leiter LA Rubino F Bhatt DL. Metabolic surgery. J Am Coll Cardiol. 2018;71:670–87. 29420964 10.1016/j.jacc.2017.12.014

[35] Kowalski GM Bruce CR. The regulation of glucose metabolism: implications and considerations for the assessment of glucose homeostasis in rodents. Am J Physiol Endocrinol Metab. 2014;307:E859–71. 25205823 10.1152/ajpendo.00165.2014

[36] Gray LR Sultana MR Rauckhorst AJ Oonthonpan L Tompkins SC Sharma A. Hepatic mitochondrial pyruvate carrier 1 is required for efficient regulation of gluconeogenesis and whole-body glucose homeostasis. Cell Metab. 2015;22:669–81. 26344103 10.1016/j.cmet.2015.07.027 PMC4754674

[37] Can E Bastiaansen JAM Couturier DL Gruetter R Yoshihara HAI Comment A. [13C] bicarbonate labelled from hyperpolarized [1-13C] pyruvate is an in vivo marker of hepatic gluconeogenesis in fasted state. Commun Biol. 2022;5:10. 35013537 10.1038/s42003-021-02978-2 PMC8748681

[38] Mosser DM Edwards JP. Exploring the full spectrum of macrophage activation. Nat Rev Immunol. 2008;8:958–69. 19029990 10.1038/nri2448 PMC2724991

[39] Hou J Zhang J Cui P Zhou Y Liu C Wu X. TREM2 sustains macrophage-hepatocyte metabolic coordination in nonalcoholic fatty liver disease and sepsis. J Clin Invest. 2021;131:e135197. 33586673 10.1172/JCI135197 PMC7880419

[40] Beljaars L Schippers M Reker-Smit C Martinez FO Helming L Poelstra K. Hepatic localization of macrophage phenotypes during fibrogenesis and resolution of fibrosis in mice and humans. Front Immunol. 2014;5:430. 25250030 10.3389/fimmu.2014.00430 PMC4157549

[41] Song E Ouyang N Hörbelt M Antus B Wang M Exton MS. Influence of alternatively and classically activated macrophages on fibrogenic activities of human fibroblasts. Cell Immunol. 2000;204:19–28. 11006014 10.1006/cimm.2000.1687

[42] Liaskou E Zimmermann HW Li KK Oo YH Suresh S Stamataki Z. Monocyte subsets in human liver disease show distinct phenotypic and functional characteristics. Hepatol Baltim Md. 2013;57:385–98. 10.1002/hep.26016 PMC4194426 22911542

[43] Tacke F Zimmermann HW. Macrophage heterogeneity in liver injury and fibrosis. J Hepatol. 2014;60:1090–6. 24412603 10.1016/j.jhep.2013.12.025

[44] Gibbons MA MacKinnon AC Ramachandran P Dhaliwal K Duffin R Phythian-Adams AT. Ly6Chi monocytes direct alternatively activated profibrotic macrophage regulation of lung fibrosis. Am J Respir Crit Care Med. 2011;184:569–81. 21680953 10.1164/rccm.201010-1719OC

[45] Idrovo JP Yang WL Jacob A Corbo L Nicastro J Coppa GF. Inhibition of lipogenesis reduces inflammation and organ injury in sepsis. J Surg Res. 2015;200:242–249. 26216747 10.1016/j.jss.2015.06.059 PMC4688159

[46] Frydrych LM Bian G Fattahi F Morris SB O’Rourke RW Lumeng CN. GM-CSF administration improves defects in innate immunity and sepsis survival in obese diabetic mice. J Immunol. 2019;202:931–42. 30578307 10.4049/jimmunol.1800713

[47] Evans L Rhodes A Alhazzani W Antonelli M Coopersmith CM French C. Surviving sepsis campaign: international guidelines for management of sepsis and septic shock 2021. Intensive Care Med. 2021;47:1181–247. 34599691 10.1007/s00134-021-06506-y PMC8486643

[48] Hauschildt KE Seigworth C Kamphuis LA Hough CL Moss M McPeake JM. Financial toxicity after acute respiratory distress syndrome: a National Qualitative Cohort Study. Crit Care Med. 2020;48:1103–10. 32697479 10.1097/CCM.0000000000004378 PMC7387748

