# Supplementary material for: Pneumosepsis survival in the setting of obesity leads to persistent steatohepatitis and metabolic dysfunction
Source: Hepatol Commun. 2023 Aug 9;7(9):e0210. doi: 10.1097/HC9.0000000000000210 (PMC10412436; doi:10.1097/HC9.0000000000000210)
Supplement: Supplementary file 1 [file hc9-7-e0210-s001.pdf]

qPCR Primer	Exon Location	Manufacturer	Assay Name
Chil3 (YM1)	3 to 4	IDT	Mm.PT.58.12585703
Col3a1	1 to 2	IDT	Mm.PT.58.13848686
β-Actin	5 to 6	IDT	Mm.PT.58. 32716599.g
