# Supplementary material for: Pneumosepsis survival in the setting of obesity leads to persistent steatohepatitis and metabolic dysfunction
Source: Hepatol Commun. 2023 Aug 9;7(9):e0210. doi: 10.1097/HC9.0000000000000210 (PMC10412436; doi:10.1097/HC9.0000000000000210)
Supplement: Supplementary file 2 [file hc9-7-e0210-s002.pdf]

	Number of hospitalizations
2015-2018 HAPPI Cohort Severe Sepsis/Septic Shock	81,196
ALT and Total Bilirubin measured in the 180 days prior to ED presentation	76,708
Weight measured in the 30 days prior to ED presentation	70,223
Weight is greater than 75 pounds and less than 500 pounds	69,845
Height measured in the 730 days prior to ED presentation	66,506
Height is less than 7 feet (84 inches) and greater than 4 feet 2 inches (50 inches)	66,136
BMI greater than or equal to 14	65,642
Baseline Total Bilirubin (lowest in 180 days prior to ED presentation is less than 4.5)	65,139
