# Supplementary figures and images for: Pneumosepsis survival in the setting of obesity leads to persistent steatohepatitis and metabolic dysfunction
Source: Hepatol Commun. 2023 Aug 9;7(9):e0210. doi: 10.1097/HC9.0000000000000210 (PMC10412436; doi:10.1097/HC9.0000000000000210)

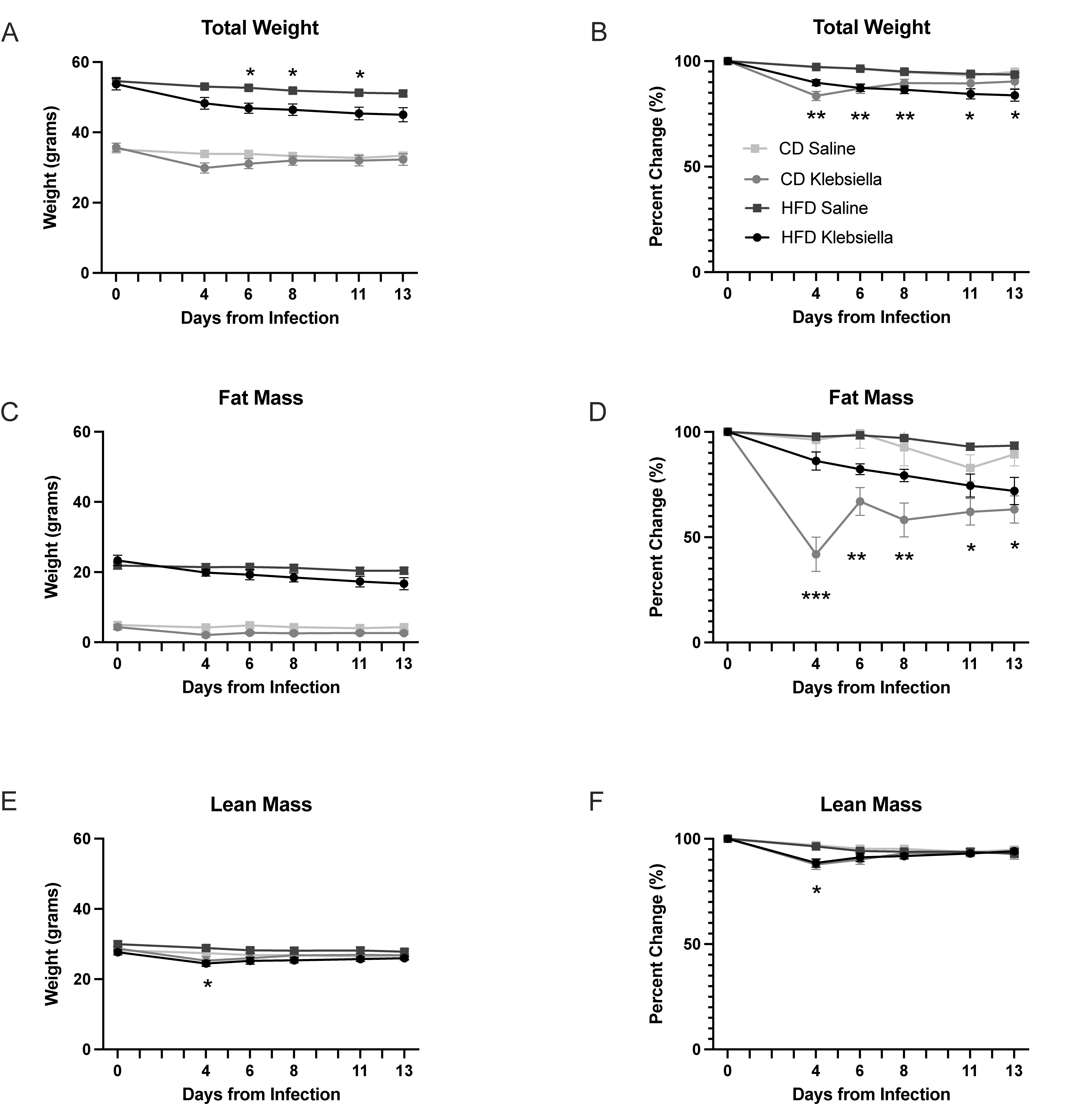

Supplement: Supplementary file 5 [file hc9-7-e0210-s005.tif]

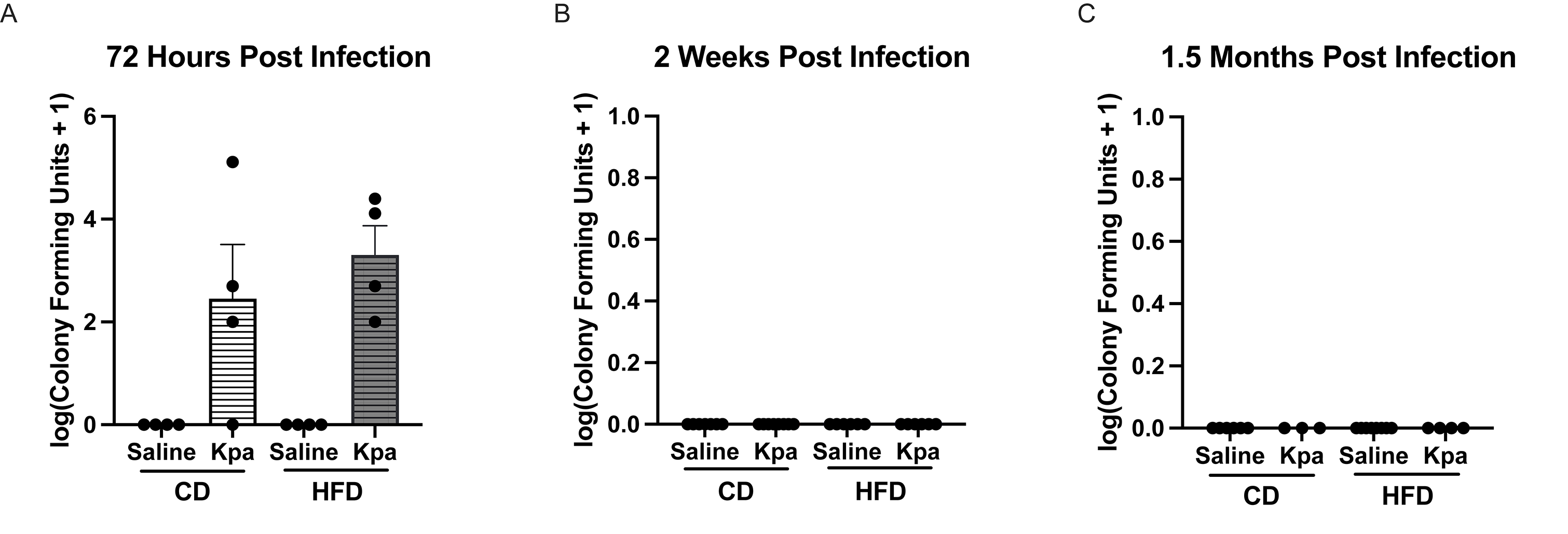

Supplement: Supplementary file 6 [file hc9-7-e0210-s006.tif]

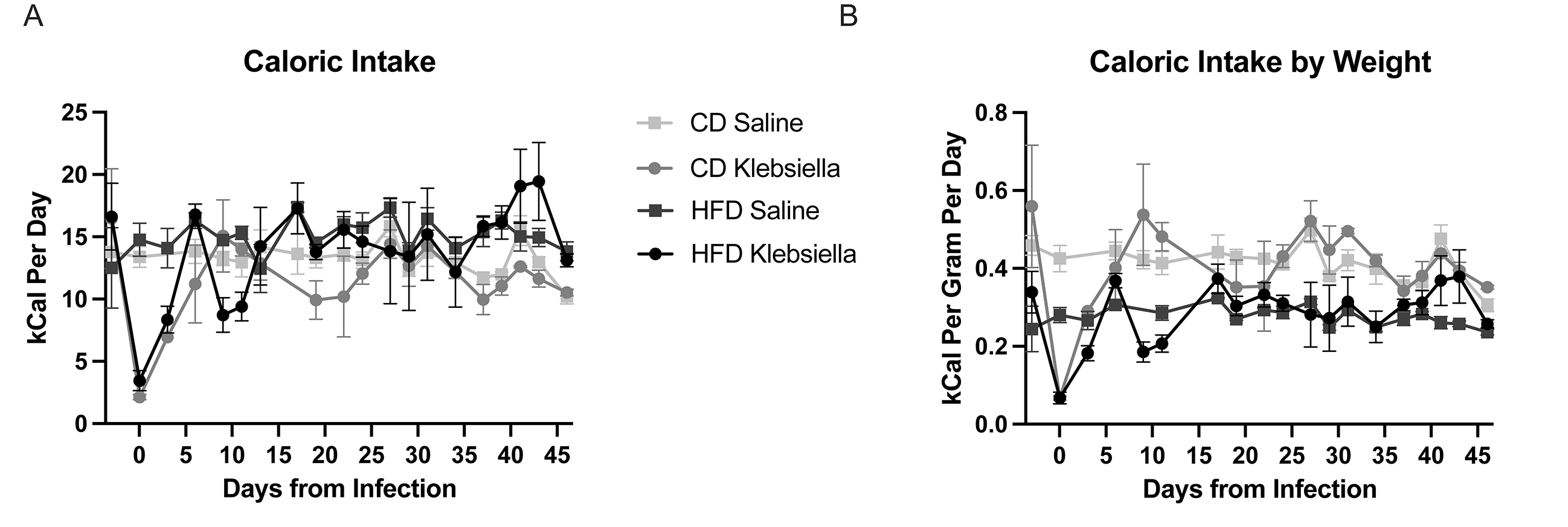

Supplement: Supplementary file 7 [file hc9-7-e0210-s007.tif]

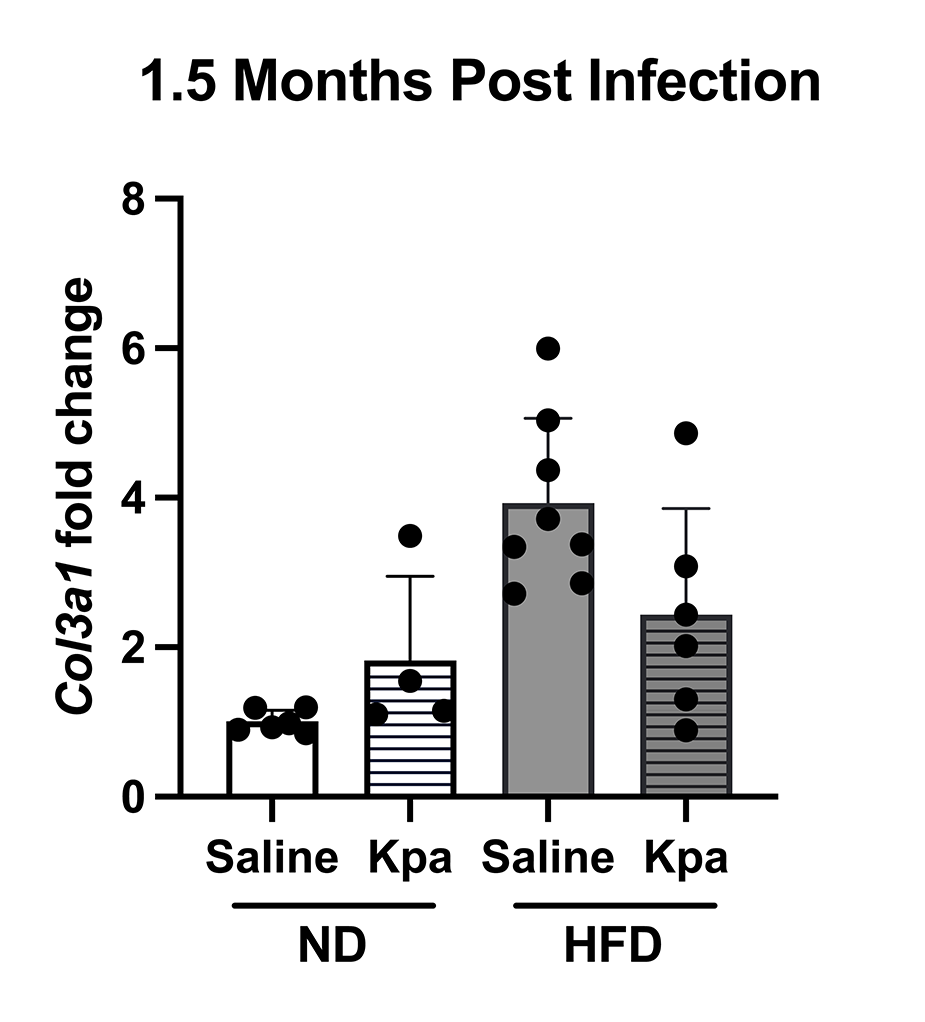

Supplement: Supplementary file 8 [file hc9-7-e0210-s008.tif]

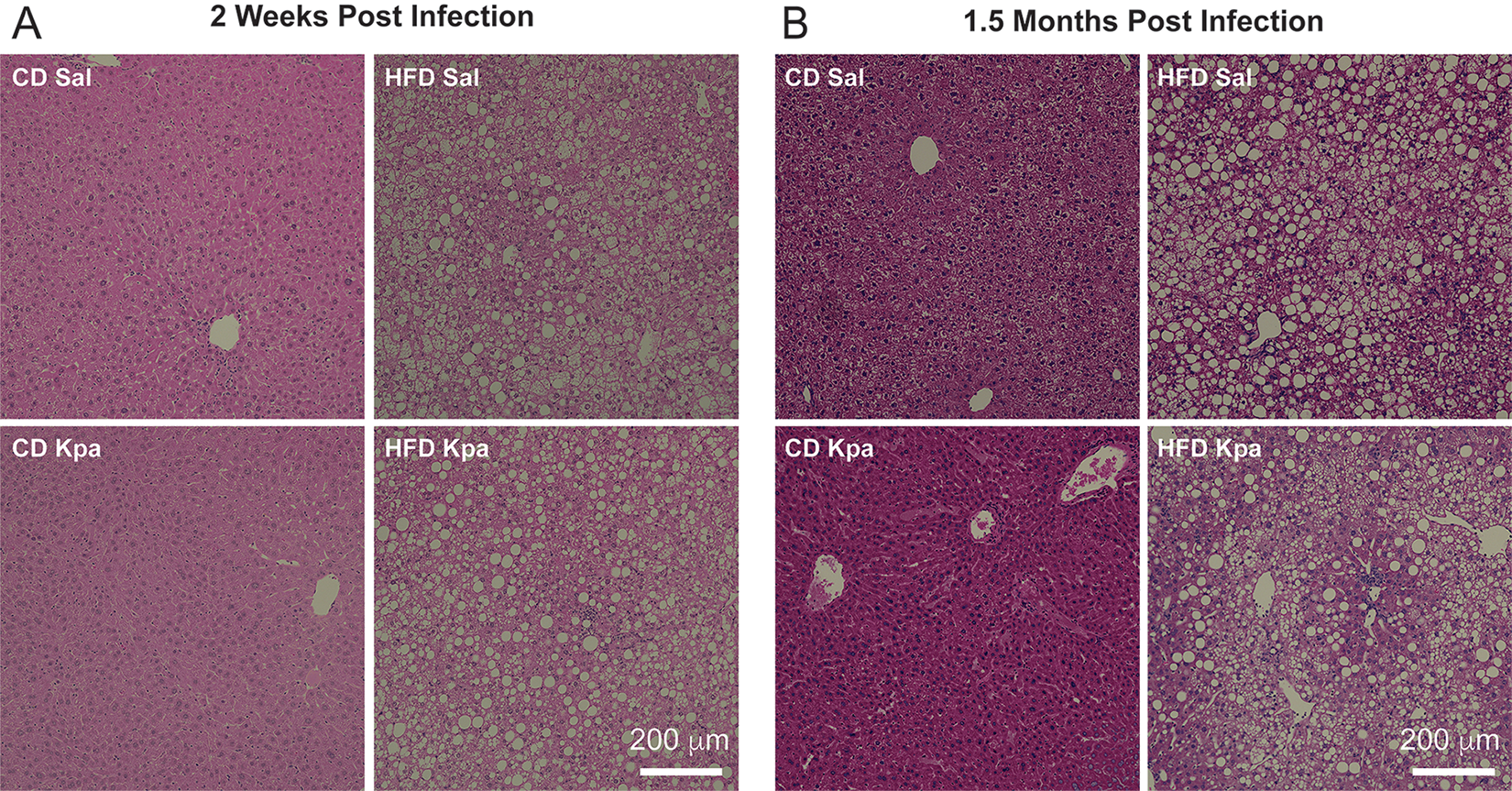

Supplement: Supplementary file 9 [file hc9-7-e0210-s009.tif]
